# Awareness of HPV and HPV vaccine among women with high-risk HPV types in the Mediterranean region

**DOI:** 10.3389/fmed.2026.1743457

**Published:** 2026-02-17

**Authors:** Serhan Can İşcan, Ufuk Atlıhan, Gökçe İşcan, Selçuk Erkılınç

**Affiliations:** 1 Departmant of Gynecology, Kütahya Health Sciences University, Kütahya, Türkiye; 2 Departmant of Gynecology, Buca Seyfi Demirsoy Training and Research Hospital, İzmir Democracy University, İzmir, Türkiye; 3 Faculty of Medicine, İzmir Democracy University, Karabağlar, Türkiye

**Keywords:** cervical cancer, high-risk HPV, HPV, HPV vaccine, human papillomavirus

## Abstract

**Aim:**

To evaluate awareness of HPV and the HPV vaccine among women with high-risk HPV types in the Mediterranean region.

**Methods:**

This observational cross-sectional study included 1,418 women who underwent colposcopy due to high-risk HPV positivity. Seven hundred seventy-six participants who fully completed the survey and provided all requisite data for the study were assessed. A survey containing 9 questions about HPV, HPV vaccine, and the source of information of HPV, relationship between HPV and cervical cancer was applied to patients who were given a colposcopy appointment due to high-risk HPV positivity.

**Results:**

Seventy percent of women underwent an HPV test during a visit to the doctor, while the proportion of those tested for screening purposes was identified as 22.2%. About 79.5% of participants reported lacking any awareness of HPV before undergoing the test. The source of their initial information of HPV, 49.2% of participants indicated health experts, while 31.4% cited social media. The percentage of participants informed about the HPV vaccine is 20.7%. Only 8.9% of participants reported receiving vaccinations.

**Conclusion:**

The fact that even women with a positive HPV test who are referred to the gynecological oncology clinic and who are in risk groups for cervical cancer have limited knowledge about HPV and the HPV vaccine shows how important it is to raise awareness in society about this issue. The use of social media, particularly the creation of content by healthcare professionals, appears to be a crucial measure for raising awareness of HPV and the HPV vaccine.

## Introduction

HPV, which causes cancer, is the second most commonly detected pathogen after *H. pylori* bacteria (1). HPV is responsible for 4.5% of all cancers in both men and women globally, being an exceedingly prevalent sexually transmitted disease. An estimated 85–90% of sexually active men and women will be infected at some point in their lifetime (2). More than 90% of HPV-related malignancies in females are cervical cancer. With almost 350,000 deaths globally in 2022, cervical cancer is ranked as the fourth most common cause of cancer and cancer-related deaths among women (3). The International Agency for Research on Cancer has identified 13 HPV types known to cause cervical cancer, with at least one type being associated with cancers of the vulva, vagina, penis, anus, and particular head and neck malignancies (4). The HPV vaccine is quite effective in preventing over 90% of malignancies caused by HPV. The prevalence of genital warts is decreasing among vaccinated teenagers and young adults. Additionally, the incidence of cervical precancer among young women has reduced due to HPV vaccination. Even though 99% of cervical cancer is caused by HPV and can be prevented by HPV vaccination, there is a significant lack of information about HPV and its vaccine (5). This has resulted in low screening rates for cervical cancer and low vaccination rates in seven developed countries.

WHO is focused on increasing public access to information on HPV, HPV vaccines, benefits, regular screening, early diagnosis, and prognosis treatment in early stages (6–8). The objective is to reduce the occurrence of cervical cancer by 2030 dramatically. Raising awareness is the first step toward achieving this objective. In the study conducted by Dillard et al., HPV awareness levels varied significantly, ranging from 10 to 95% (9). Similar variability has been reported in HPV vaccine awareness, ranging from 63% among American males (10) to 87% in the general population (11) to over 95% among female university students (12).

According to the “Health Belief Model” individuals’ health behaviors are influenced by their beliefs, values, and attitudes. If these problematic beliefs and attitudes are identified, the health education or treatment methods to be applied can be determined in a way that is more appropriate for that person (13).

Individuals are more likely to engage in preventive behaviors when they perceive themselves to be at risk, comprehend the severity of the disease, recognize the advantages of preventive measures, and receive appropriate instructions to initiate action. In the current study, despite being HPV positive and consequently objectively at high risk, the majority of participants demonstrated a dearth of awareness regarding HPV, its association with cervical cancer, and the protective function of the vaccine; this highlights a significant deficiency in perceived awareness and perceived severity.

Many previous articles have questioned the relationship between HPV awareness and race, social status, and education level. This study investigated the awareness of HPV and the HPV vaccine, the sources of information about HPV and the HPV vaccine, and the reasons for undergoing HPV testing in all women with high-risk HPV positivity regardless their educational backgrounds and socioeconomic statuses before colposcopic evaluation and detailed information and counseling by gynecological oncologists and their team. As these women are at high risk of cervical cancer compared to the general population, it is essential to know their level of awareness about HPV and the HPV vaccine.

## Materials and methods

**FIGURE 1:**
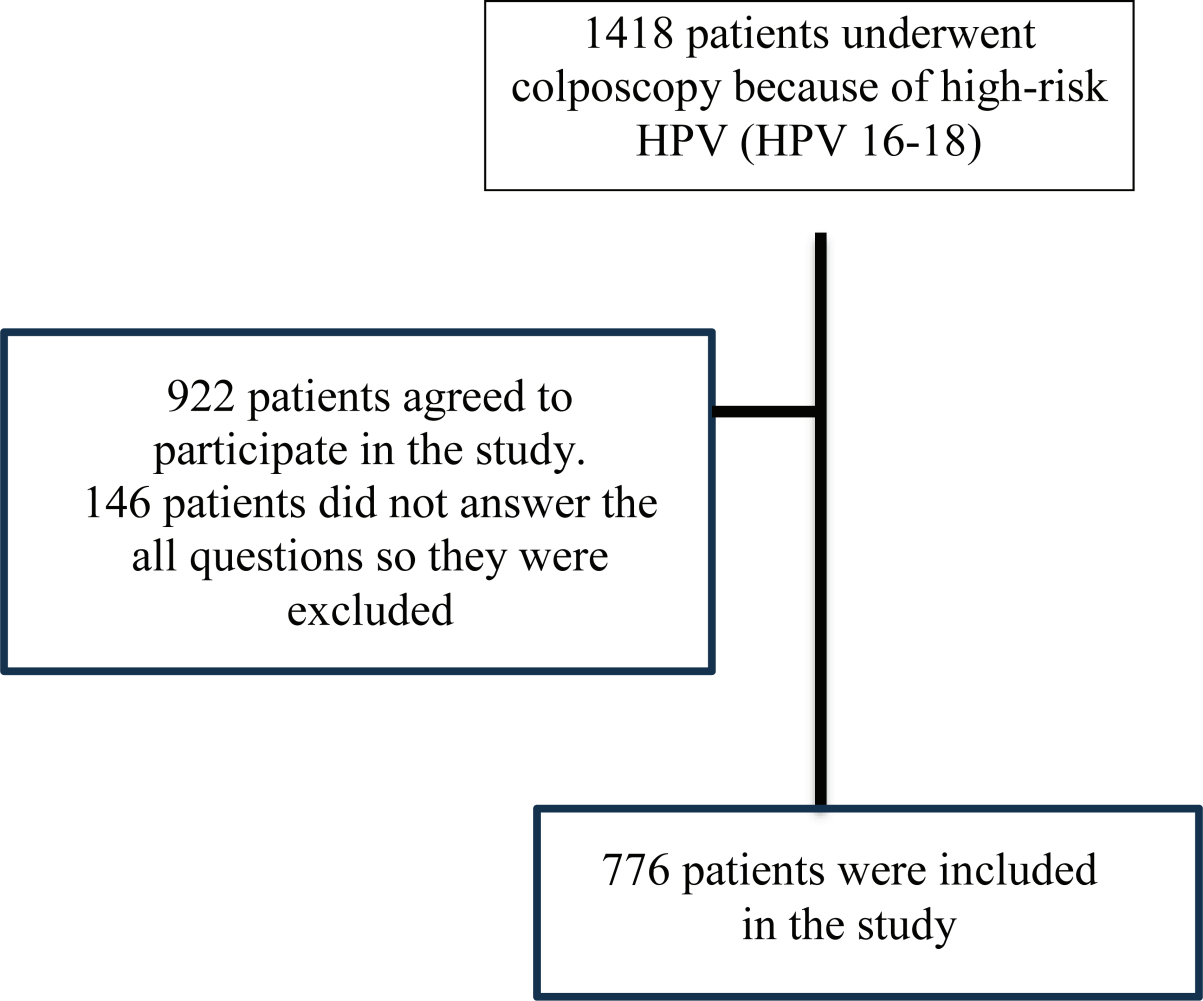
Patient selection flowchart.

This is a cross-sectional observational survey-based study. The research received clearance from the Suleyman Demirel University Health Sciences Ethics Committee (approval no. 06-01-2025, 88, 14). Subsequent to ethical committee permission, colposcopy was performed on 1,418 women identified as high-risk HPV (HPV 16–18) positive and referred to the gynecological oncology department. Prior to the colposcopy, all women, without exception, were administered a 9-question survey. Out of these women, 922 agreed to participate in the study. Following the exclusion of women with incomplete characteristics or responses, the analysis was conducted on the data of 776 women with complete information (Figure 1).

A survey containing 9 questions about HPV, HPV vaccine, and the source of information on HPV, and the relationship between HPV and cervical cancer was included in the survey. Besides awareness about HPV, HPV vaccine, and cervical cancer, general information like age, height, weight, parity, and lifestyle habits like smoking and alcohol use was obtained. The impact of alcohol consumption, smoking, and parity on awareness of HPV and the HPV vaccine was examined utilizing the chi-square test. The patients’ ages were classified according to generational age categories: Baby Boomers (Born 1946–1964), Generation X (Born 1965–1980), Millennials or Generation Y (Born 1981–1996), Generation Z (Born 1997–2010). To determine whether there was a linear association between generational age categories and the HPV vaccine and HPV awareness, *P-*values for trend were calculated by treating generational categories as a continuous variable. The relationship between BMI index categories and awareness of HPV and the HPV vaccine was similarly investigated.

Descriptive statistics, including means, standard deviations, frequencies, and percentages, were utilized to summarize the data. A crosstab analysis was performed to examine the relationships among category variables. The Mann-Whitney U test was employed to assess differences between two groups, and the Kruskal-Wallis test was used to determine differences among independent groups for continuous variables that are not regularly distributed. A binary logistic regression analysis was conducted to identify factors associated with HPV vaccination status. The variables included in the final model were age, HPV vaccine awareness, and access to information. Regression coefficients (B), standard errors (S.E.), Wald test statistics, significance values (p), probability ratios [Exp(B)], and 95% confidence intervals were reported. A step-by-step evaluation was conducted to assess model suitability, and the final model was selected based on significance levels and explanatory power. A significance level of *p* < 0.05 was deemed statistically significant. All analyses were performed utilizing statistical software (SPSS).

## Results

The study comprised 776 women who had a colposcopic evaluation due to high-risk HPV positivity. The age range of women in this study is 21–75, with a mean age of 44. The demographic features of women presented in Table 1 indicate that 31.2% were smokers and 9.4% were alcohol consumers. Analysis of body mass index (BMI) averages indicates that the majority of individuals fall within the normal or overweight categories (Table 1).

**TABLE 1:** Demographic characteristics of the participants.

Variables	n	%
**Generation**		
Baby boomers (1946–1964)	69	8.9
Generation X (1965–1980)	301	38.8
Millennials or generation Y (1981–1996)	333	42.9
Generation Z (1997–2010)	73	9, 4
**BMI**		
Underweight ( < 18, 5)	15	1.9
Normal weight (18, 5–24, 9)	258	33.2
Overweight (25–29, 9)	310	39.9
Obese ( ≥ 30)	193	24.9
**Parity**		
0	85	11.0
1	124	16.0
≥ 2	567	73.1
**Smoking**		
Yes	242	31, 2
No	534	68, 8
**Alcohol intake**		
Yes	73	9, 4
No	703	90, 6
Total	776	100

### HPV testing characteristics and baseline HPV awareness

A 70% of women underwent an HPV test during a visit to the doctor, while the proportion of those tested for screening purposes was identified as 22.2%. A total of 79% participants reported lacking any awareness of HPV before undergoing the test. The percentage of those aware that HPV is a sexually transmitted virus is 33.1%. Just over one-third of the population (33, 5%) is aware that human papillomavirus (HPV) can cause cervical cancer.

When inquired about the source of their initial information of HPV, 49.2% of participants indicated health experts, while 31.4% cited social media (Table 2).

**TABLE 2:** Distribution of responses related to HPV awareness.

Variables	n	%
**1. For what reason was the HPV test performed?**		
During the examination	546	70.4
National cervical screening program	172	22.2
Recommendation from a friend, neighbor, etc.	9	1.2
Social media	30	3.9
Other reasons	19	2.4
**2. Ahead of this test, were you aware of HPV?**		
Yes	159	20.5
No	617	79.5
**3. Did you know that HPV is a sexually transmitted virus?**		
Yes	257	33.1
No	519	66.9
**4. Did you know that HPV is associated with cervical cancer?**		
Yes	260	33.5
No	516	66.5
**5. Where did you get the first information about HPV?**		
Social media	244	31.4
Social interaction (Friends, etc.)	90	11.6
Family	14	1.8
Healthcare professionals (Doctor, nurse, etc.)	382	49.2
Others	46	5.9

### HPV vaccine awareness and vaccination status

The percentage of participants who were informed about the HPV vaccine is 20.7%. The majority of participants (61.1%) reported receiving information about the HPV vaccine from healthcare professionals, including nurses and doctors, during their colposcopy appointments, whereas 20.1% acquired it through social media. Only 8.9% of participants reported receiving vaccinations. The percentage of the population who contemplated vaccinating their children after being informed about the vaccine was merely 6.1% (Table 3).

**TABLE 3:** Distribution of responses related to HPV vaccine awareness.

Variables	n	%
**6. Do you have information about the HPV vaccine?**		
Yes	161	20.7
No	615	79.3
**7. Where did you get the first information about the HPV vaccine?**		
Social media	156	20.1
Social interaction (Friends, etc.)	86	11.1
Family	13	1.7
Healthcare professionals (Doctor, nurse, etc.)	474	61.1
Others	47	6.1
**8. Have you been vaccinated against HPV?**		
Yes	69	8.9
No	707	91.1
**9. Having acquired awareness on the HPV vaccine, would you contemplate vaccinating your child?**		
Yes	47	6.1
No	729	93.9

### Factors associated with HPV vaccine awareness

Table 2 presents factors associated with awareness of HPV vaccination. There were statistically significant differences in HPV vaccine awareness according to age groups (*p* < 0.001), parity (*p* < 0.001), reason for HPV testing (*p* = 0.001), and knowledge of the relationship between HPV and cervical cancer (*p* < 0.001). Awareness of HPV vaccination was higher among participants who knew the association between HPV and cervical cancer.

### According to age groups, body mass index), and parity

Millennials are the most aware generation regarding the HPV vaccine. Generation Z showed greater awareness than older age groups. Generation X and baby boomers exhibit indifference regarding vaccination (*p* < 0.001). HPV vaccine awareness demonstrated a decreasing trend with increasing age (Table 4). Generation Z and millennials acquired information about HPV through social media, but Generation X sourced it from healthcare professionals (*p* < 0.001). For baby boomers, the primary sources of awareness regarding the HPV vaccine were friends and health care professionals.

**TABLE 4:** Factors associated with awareness of HPV vaccination.

Variable	No awareness vaccination n(%)	Awareness vaccination n(%)	*P*-value
Age groups			< 0.001
Generation z	48 (7.8)	25 (15.7)	< 0.001
Millennials	252 (40.8)	81 (50.9)	< 0.001
Generation x	254 (41.2)	47 (29.6)	< 0.001
Baby boomer	63 (10.2)	6 (3.8)	< 0.001
**Body mass index (BMI)**			
Underweight	11 (1.8)	4 (2.5)	0.043
Normal weight	193 (31.2)	65 (40.9)	0.043
Over weight	248 (40.2)	62 (39)	0.043
Obese	165 (26.7)	28 (17.6)	0.043
**Parity group**			
No child	56 (9.1)	29 (18.2)	< 0.001
One and more children	561 (90.9)	130 (81.8)	< 0.001
**Why did you give the HPV test?**			
Examination	447 (72.7)	99 (61.5)	0.001
Screening	132 (21.5)	40 (24.8)	0.001
Friends etc.	8 (1.3)	1 (0.6)	0.001
Social media	18 (2.9)	12 (7.5)	0.001
Other	10 (1.6)	9 (5.6)	0.001
**Do you know the relation between HPV and cervix cancer?**			
No	493 (78.5)	33 (20.5)	< 0.001
Yes	132 (21.5)	128 (79.5)	< 0.001

Values are presented as n (%). *P*-values were calculated using the chi-square test.

In contrast, Generation X predominantly relied on health care professionals for this information, as they had done for HPV knowledge (*p* < 0.001). Millennials are the most aware generation regarding the HPV vaccine. Generation X and baby boomers exhibit indifference regarding vaccination (*p* < 0.001).

HPV vaccine awareness differed significantly across body mass index categories (*p* = 0.043). Participants with normal body weight showed greater awareness of the HPV vaccine than obese individuals. HPV vaccine awareness tended to decrease with increasing BMI (Table 4).

Participants without children showed greater awareness of the HPV vaccine than those with one or more children (*p* < 0.001). HPV vaccine awareness was inversely associated with parity (Table 4).

### Multivariable analysis of factors associated with HPV vaccination

A backward stepwise logistic regression analysis was performed to identify independent factors associated with HPV vaccination status. Variables of clinical significance and those found significant in univariate analyses were included in the initial model. Backward elimination (Wald) was applied. With an exclusion criterion of *p* > 0.10. Odds ratios (OR) were reported with 95% confidence intervals. Model fit was assessed using the Hosmer-Lemeshow goodness-of-fit test. The goodness-of-fit of the final backward logistic regression model was acceptable, as indicated by a non-significant Hosmer–Lemeshow test (χ^2^ = 5.98, df = 8, *p* = 0.649).

First of all. The variable answering the question “Where did you get the information about the vaccine?.” Which initially consisted of five categories, was grouped into three conceptually meaningful categories to improve the stability and interpretability of the model as age increases. The probability of getting the HPV vaccine decreases statistically significantly (OR = 0.858; 95% CI = 0.825–0.893; *p* < 0.001). Each age group increase reduces the likelihood of vaccination by about 14%. A participant’s knowledge about the HPV vaccine increases their likelihood of getting vaccinated by approximately 12.6 times. This is a powerful and significant effect (OR = 12.635; 95% CI = 6.642–24.034; *p* < 0.001) (Table 5).

**TABLE 5:** Determination of factors affecting HPV vaccination status (backward logistic regression).

Variables	OR	95% CI	*P*
Age	0.858	0.825–0.893	< 0.001
Do you have information about the HPV vaccine?	12.635	6.642–24.034	< 0.001
Where did you get the first information about the HPV vaccine?	1.565	0.355–6.904	0.350
Where did you get the first information about the HPV vaccine? (Healthcare professionals)	−	−	0.001
Social media vs. Healthcare professionals	0.446	0.140–1.420	0.172
Informal vs. Healthcare professionals	0.226	0.101–0.509	< 0.001

## Discussion

Various studies indicate different levels of awareness of HPV and the HPV vaccine (6–15). In comparison to several studies in the literature, our research, despite focusing on high-risk HPV-positive women, revealed alarmingly low awareness rates. Low awareness may contribute to reduced uptake of preventive behaviors; however, causality cannot be inferred. Furthermore, it is essential to explore the reasons for their lack of information regarding this issue, despite being HPV positive.

Researches have shown that smoking and alcohol consumption elevate the incidence of HPV and cervical intraepithelial lesions (16–19). HPV clearance is prolonged in smokers as well (20). The rate of smoking and alcohol consumption in the patients in our study was observed to be low. No difference was found in the awareness of HPV and the HPV vaccine between patients who smoked and those who did not.

Obesity is acknowledged as an impediment to accessing women’s preventative health treatments, such as cervical and breast cancer screenings. Women with obesity may experience increased feelings of isolation and may be perceived as less attractive; previous researches have indicated that women with obesity are less likely to be sexually active, but if they are sexually active, they are more prone to participate in high-risk sexual practices. Obese girls and women exhibited a lower likelihood of reporting HPV vaccination and, when vaccinated, received the immunization at an older age (21). In other research, a higher body mass index (BMI) is not correlated with decreased initiation or completion of the HPV vaccine series, as well as the age at which the three-dose regimen is initiated among a general population sample of US teenagers (22). Our research revealed that participants in the normal weight category exhibit greater awareness of HPV compared to other categories. This result can be attributed to the fact that people with normal weight are more sensitive and conscious about their health.

Despite the utilization of social media prevalent across all educational and social strata globally for informational purposes, prior publications addressing race, social position, and educational attainment had not thoroughly examined the effect of social media. There are some studies indicating that information regarding HPV and the HPV vaccine is acquired via social media, leading to heightened awareness; these studies demonstrate that such awareness does not influence vaccination rates (14). This study, in contrast to others, interrogates the understanding of HPV and the HPV vaccine, highlighting the limited utilization of social media in health education. This may elucidate the scarcity of information, especially among HPV-positive women. Although social media is part of our lives, people prefer to receive health information from health professionals, and their guidance is effective in this regard (6, 23). Intensive use of social media may not provide information about health and HPV. This suggests that a lack of high-quality information on social media may hinder HPV awareness. Especially considering the elderly, men, low-income people, those with a low level of education, and those who cannot use the national language sufficiently, preparing social media content using techniques such as storytelling and infographics can increase awareness about HPV and the HPV vaccine (24). This study reveals that awareness of HPV and cervical cancer is inadequate, and the understanding and vaccination rates about the HPV vaccine are poor. Enhancing informational and awareness initiatives by healthcare professionals, alongside national screening and vaccination programs, can significantly contribute to the prevention of HPV (5, 22–26).

Numerous papers indicate that awareness of HPV and the HPV vaccine is greater among younger individuals (1, 6, 24); yet, some studies suggest that age does not influence this awareness (9–12). In this study, Generation X had an HPV test with the guidance of social media, but acquired information about HPV from a health care professional. Generation Z and millennials acquired information about HPV through social media, and millennials are the most aware generation regarding the HPV vaccine. The most vulnerable group to HPV is the baby boomer generation, who are frequently unaware that HPV is a sexually transmitted infection. This suggests that the older generation does not sufficiently educate younger generations, and may indeed contribute to the poor awareness observed in the study.

An essential consequence of information regarding HPV and the HPV vaccine is that it empowers individuals to adopt preventive measures against diseases associated with HPV. When parents possess information regarding HPV and the HPV vaccine, both they and their children have higher vaccination rates. One detail here is that the families accept the vaccine price. The more affordable the price is for the family, the higher the vaccination rate (27). In our study, patients without children had statistically more information about HPV and the HPV vaccine than those with children (*p* < 0.01). Women without children are younger than those with children, and they predominantly identified social media as their primary source of information. HPV tests were typically administered to patients with children during cervical cancer screenings, and they also received information about HPV from healthcare experts. Patients without children typically underwent HPV testing during their examination. Our survey revealed a significantly low level of awareness regarding HPV and its vaccine. Consequently, vaccination rates and the inclination to vaccinate their children are markedly low. Research indicates that parental intention to vaccinate their children against HPV correlates with awareness of HPV. Notwithstanding the significance of HPV awareness, studies reveal that individuals possess a limited understanding of HPV. Recommendations or information from health care experts regarding HPV is essential to enhance awareness of the virus (24).

The influence of social media is indisputable; numerous contents educate society on healthy living and provide guidance. Even in studies where the majority of participants had significant awareness and engagement in positive behaviors related to cervical cancer prevention, HPV awareness increased with short-term involvement in an online social media platform and receipt of personalized health messages (28). Compared with nonusers, participation in one, two, three, or four social media activities is associated with increased HPV awareness. Heightened social media engagement correlates with enhanced awareness of HPV and the HPV vaccine among adults residing with children. Social media initiatives might enhance awareness of the cancer prevention advantages of the HPV vaccination, perhaps mitigating HPV-vaccine hesitancy and promoting vaccine uptake to decrease cancer incidence rates among at-risk populations (29, 30). Developing additional institutional and governmental informational resources on this topic will also mitigate the dissemination of misinformation (31, 32). This study highlights the need to assess individuals’ awareness levels and health beliefs, as well as to formulate personalized recommendations based on the findings.

Cervical cancer, while not a significant health burden in Turkey, has been subjected to screening using cervical smear tests since 1992, following the guidelines of the World Health Organization (WHO). This smear-based screening, conducted for over 20 years, has significantly underachieved the target of 70% coverage due to various factors, including reliance on specialists, their indifference toward the issue, and insufficient public awareness. Nonetheless, hardly 20% of the intended female population has undergone screening as part of the initiative. According to the national cancer screening criteria revised in 2014, all women aged 30–65 are subjected to HPV testing every 5 years, with positive results subsequently assessed through a smear test. HPV screening programs and vaccination are demonstrably successful in preventing cervical cancer; yet, sufficient rates of screening and vaccination have not been attained even in developed societies (1, 5, 17, 18, 33). The leading cause for this is the inadequate awareness of HPV and the HPV vaccine. For instance, even in the United States in 2020, 64% of individuals recognized HPV, and 60% were familiar with the HPV vaccine (34). Furthermore, vaccination rates in developing countries, such as Türkiye, have remained persistently lower due to insufficient awareness of vaccination, the prohibitive cost of vaccines, and inadequate governmental support for immunization initiatives (14, 35). A notable finding in our study is the low rate of vaccination among individuals and their children following the acquisition of information regarding the HPV vaccine. We contend that the justifications for this predicament warrant thorough examination. Facilitating complementary access to the vaccine through national vaccination initiatives may enhance these rates. The fact that even women with a positive HPV test who are referred to the gynecological oncology clinic and who are in risk groups for cervical cancer have limited awareness about HPV and the HPV vaccine shows how important it is to raise awareness in society about this issue. The study has limitations, including a single-center design, self-reported vaccination status, potential response bias, and the timing of counseling. The use of social media, particularly the creation of content by healthcare professionals, appears to be a crucial measure for raising awareness of HPV and the HPV vaccine. In developing comprehensive programs and national initiatives for HPV and cervical cancer, it is essential to acknowledge the societal lack of awareness of this issue and to organize the measures properly to facilitate significant advancement.

## Data Availability

The raw data supporting the conclusions of this article will be made available by the authors, without undue reservation.
